# Early adaptation to high‐altitude: Mood and cognitive responses at simulated 4500 m

**DOI:** 10.14814/phy2.70601

**Published:** 2025-10-08

**Authors:** Ricardo Muller Bottura, Ambra Bisio, Debora Cristina Hipolide

**Affiliations:** ^1^ Department of Psychobiology Universidade Federal de São Paulo São Paulo Brazil; ^2^ Department of Neurosciences, Biomedicine and Movement Sciences Università degli Studi di Verona Verona Italy; ^3^ Department of Neuroscience, Rehabilitation, Ophthalmology, Genetics and Maternal and Child Sciences (DINOGMI) Università degli Studi di Genova Genova Italy; ^4^ Department of Experimental Medicine (DIMES) Università degli Studi di Genova Genova Italy; ^5^ Centro Polifunzionale di Scienze Motorie Università degli Studi di Genova Genoa Italy

**Keywords:** acclimatization, heart rate, hypoxia, mountain sickness, oxygen saturation

## Abstract

High‐altitude exposure induces hypoxemia, triggering metabolic adjustments to enhance oxygen delivery. This acclimatization process often involves mood changes, cognitive alterations, and Acute Mountain Sickness (AMS) symptoms. This study examined the effects of simulated hypoxia at 4500 m on physiological variables, reaction time, lapses, and mood states. Twenty‐three volunteers were exposed to a simulated 4500 m altitude for 4 h in a normobaric hypoxia chamber. Lactate, glucose, SpO_2_, heart rate (HR), and AMS symptoms were assessed before and every hour during exposure. As expected, SpO_2_ significantly decreased (*p* < 0.001), while HR increased (*p* = 0.006) during exposure. Reaction time remained unaffected, but lapses significantly correlated with lactate levels (*p* = 0.002). Mood states, particularly tension (*p* = 0.001) and fatigue (*p* < 0.001), were associated with AMS symptoms. Simulated high‐altitude exposure induced physiological alterations, including decreased SpO_2_, increased HR, and a significant correlation between lactate levels and cognitive performance. While reaction time was unchanged, mood disturbances were strongly related to AMS. These findings highlight the need for strategies to mitigate both physiological and psychological effects of hypoxia.

## 1 INTRODUCTION

Acute Mountain Sickness (AMS) has traditionally been attributed to decreased arterial oxygen saturation (Burtscher et al., 2011; Hsu et al., 2014; Karinen et al., 2010); however, recent evidence suggests that distal effects on cerebral hemodynamics, such as impaired autoregulation or increased intracranial pressure, may play a more central role (West, 2012), which negatively affects cognitive function (Dykiert et al., 2010) and may be related to sea‐level trait anxiety (Boos et al., 2018). AMS typically presents symptoms such as headache, dizziness, nausea, and fatigue, affecting both physical and mental performance in high‐altitude environments (Hackett & Roach, 2001).

Furthermore, cognitive function is also impacted by altitude, primarily due to hypoxemia which can lead to cerebral edema (HACE) (Lopez et al., 2013), a potentially life‐threatening condition characterized by symptoms such as dizziness, vertigo, and delayed reaction time (Carod‐Artal, 2014), thereby impairing cognitive capacity. This negative impact on cognitive function is associated with decreased arterial oxygen saturation (SaO_2_) (Ando et al., 2013).

In addition to cognitive impacts, acute hypoxic exposure can also disturb mood states. Symptoms such as euphoria, irritability, and hostility begin to manifest at altitudes of 3000 meters (m) (Bahrke & Shukitt‐Hale, 1993), with severity increasing with longer exposure or higher altitudes (Shukitt‐Hale, 1988). For example, Shukitt‐Hale et al. (1990) demonstrated that individuals climbing to an altitude of 3080 m experienced decreased vigor and increased fatigue even with a gradual ascent (<300 m/day). The physical exertion of climbing exacerbated the reduced oxygen supply caused by hypoxia, further affecting the individual's affective responses. These findings are further supported by a recent study using a hypobaric chamber simulating altitudes between 3000 and 4050 m, where decreased vigor and increased fatigue and depression were observed in individuals showing AMS symptoms (Figueiredo et al., 2022).

Given the challenges of studying hypoxia at real altitudes, technological advancements have provided alternative solutions. Normobaric and hypobaric chambers, like the one used in the latter study, have enabled researchers to simulate high‐altitude environments in controlled settings, allowing the study of the effects of hypoxia without the logistical challenges of real‐world altitude. Simulated altitude has been used effectively to investigate both cognitive impairments and mood disturbances caused by hypoxia (Figueiredo et al., 2022; Lemos et al., 2012), providing a controlled environment to isolate the effects of reduced oxygen availability. This methodology allows for examining AMS symptoms without the influence of physical exertion, as seen in real altitude exposures.

While low altitudes (1300 and 2600 m) do not typically harm cognitive function (Ando et al., 2013), higher simulated altitudes can be detrimental. For instance, exposure to a simulated altitude of 5500 m for 90 min can impair various cognitive dimensions, including increased reaction time (Dykiert et al., 2010), decreased memory, and attention, which encompass the ability to organize thoughts, prioritize tasks, and make decisions (Turner et al., 2015).

Although previous studies have explored the impact of altitude on cognitive and mood states, few have focused specifically on the initial hours of exposure (<12 h), a critical window during which AMS symptoms may emerge (Hackett & Roach, 2001) and cognitive decline can begin to manifest (Falla et al., 2021). As a result, the effects on reaction time and mood states during this early acclimatization period remain unclear. Monitoring symptom progression during the first few hours at altitude is crucial, as it can inform decisions on when to initiate therapeutic interventions, particularly since the initial symptoms of AMS can emerge within 6 h of exposure (Burtscher et al., 2014). Therefore, this study aimed to examine the impact of hypoxia on reaction time and mood states in physically active individuals during the first 4 h of exposure to a simulated altitude of 4500 m.

## 2 MATERIALS AND METHODS

### 2.1 Participants

This study was approved by the Research Ethics Committee of the Universidade Federal de São Paulo under protocol number 2.001.055 and adhered to the norms established by Brazilian Legislation in Resolution n. 466/2012 of the National Health Council. Volunteers were provided with all necessary information about the assessments and subsequently signed an Informed Consent Form (ICF). Participants under 18 years of age provided written assent, and their parents or legal guardians provided written informed consent before any study procedures. The number of participants was determined through sample size calculation using GPower 3.1 software (ANOVA Repeated measures, within factors, effect size 0.25, alpha 0.05, power 0.8, 1 group, 5 measurements. Minimum number of participants: 21). A total of 23 physically active individuals (8 females and 15 males) aged between 17 and 45 years (28.87 ± 8.20 years) participated in the study. Participants who had been exposed to altitudes above 2.500 m within 6 months before the experiment were excluded to prevent any effects of residual acclimatization.

### 2.2 Procedure

Participants were assessed at five distinct moments: (1) immediately before entering the chamber, (2) after 1 h, (3) 2 h, (4) 3 h, and (5) 4 h of hypoxia exposure. Blood samples were collected at each time point to measure lactate and glucose plasma concentrations. Heart rate (HR) and peripheral oxygen saturation (SpO2) were also measured. Additionally, the Lake Louise Questionnaire for Acute Mountain Sickness (AMS) symptoms was completed at each time point. All assessments were conducted in the morning. Participants arrived in a fasted state and, after the initial analyses (1) performed outside the chamber, they received a standardized snack.

### 2.3 Altitude normobaric chamber

The altitude simulator (normobaric chamber; CAT‐Air Unit 12, Colorado Altitude Training™, Boulder, CO, USA) simulates altitudes up to 4.500 m. The equipment consists of two air units installed outside the chamber that facilitate gas exchange by increasing nitrogen and reducing oxygen levels. This gas exchange creates a difference in oxygen concentration inside the chamber, which is displayed in real‐time on a monitor showing the simulated altitude, measured by a module containing an oxygen sensor sensitive to its variations.

### 2.4 Acute mountain sickness (AMS) symptoms

AMS was assessed using the Lake Louise Score (LLS), a self‐report questionnaire validated by Roach et al. (1993, 2018). The LLS evaluates five symptoms: headache, gastrointestinal discomfort (nausea or loss of appetite), fatigue or weakness, dizziness, and sleep disturbance, each rated on a 0–3 scale (0 = absent; 1 = mild; 2 = moderate; 3 = severe). According to diagnostic criteria, AMS is defined by the presence of at least two symptoms, one of which must be a headache, at altitudes exceeding 2.500 meters.

Although the LLS was originally designed for categorical diagnosis using symptom thresholds, in this study we treated the total LLS score as a continuous variable to quantify the severity of AMS‐related symptoms over time and to explore associations with physiological and behavioral outcomes.

### 2.5 Physiological variables

SpO_2_ and heart rate (HR) were monitored using a MedChoice MD300C202 fingertip pulse oximeter (Beijing Choice Electronic Technology Co., Ltd., Beijing, China), which uses dual light sources (660 nm red LED and 905/880 nm infrared LED) and a photodetector to detect variations in arterial blood oxygen saturation and pulse rate.

Blood samples for glucose and lactate were collected using a drop of blood obtained from the fingertips. The volunteer's fingertip was cleaned with an alcohol swab (BIOSOMA) before puncturing it with an auto‐lancet (BIOLAND Model SB‐323). The quantification of these variables was performed using reflectance photometry with the Accutrend Plus monitor (Roche Diagnostics, Mannheim, Germany), a portable analyzer for the quantitative determination of glucose, lactate, cholesterol, and triglycerides.

### 2.6 Neurobehavioral measures

#### 2.6.1 Behavioral attention

Reaction time was assessed using the Psychomotor Vigilance Test device (PVT‐192, Psychology Software Tools, Pittsburgh, PA, USA) to investigate the influence of decreased arterial oxygen pressure on this cognitive function. During the test, volunteers were instructed to pay attention to a red light, which would randomly illuminate on a rectangular display at the top of the device, and to press the button as quickly as possible once the light became visible. If volunteers took over 355 ms to press the button, the device recorded that attempt as a Lapse, and the number of Lapses was quantified. We used the 3‐min protocol (Basner & Dinges, 2011) to ensure there was enough time for each individual to perform all tasks on an hourly basis, as we had up to five volunteers participating each day of the experiment.

#### 2.6.2 Mood states

Mood states were assessed using the Brunel Mood Scale (BRUMS), originally developed by Terry et al. (2003) and validated for use in Brazilian Portuguese by Rohlfs et al. (2008). The scale comprises 24 adjectives describing mood states, each rate on a 5‐point Likert‐type scale ranging from 0 (“not at all”) to 4 (“extremely”). These items are grouped into six affective dimensions: tension, depression, anger, vigor, fatigue, and confusion. Scores for each subscale were computed by summing the four relevant items, yielding a range from 0 to 16 per dimension. No transformations or standardizations were applied; all results are presented as raw scores (mean ± SD). Notably, a higher score in the vigor dimension relative to the others is considered indicative of a healthy mood profile, commonly referred to as the “Iceberg Profile.”

### 2.7 Statistical ANALISYS

Descriptive variables of the sample were tested for normality using Shapiro–Wilk tests, due to the sample size of 23 volunteers. As the data showed normal distribution, they were presented as mean and standard deviation. The outcome variables included physiological changes in peripheral oxygen saturation (SpO_2_) and heart rate (HR), the relationship between glucose consumption, lactate production, and cognitive performance, and the association between mood states and symptoms of Acute Mountain Sickness. Repeated‐measures General Linear Models (GLM) with Bonferroni post‐hoc tests were used to assess changes in variables over time. Generalized Estimating Equations (GEE) models with a Poisson distribution were used to analyze the associations between physiological variables, reaction time, lapses, and mood states. The GEE approach was chosen because it is particularly well‐suited for repeated measures data, as it accounts for the correlation between measurements taken from the same individual over time. Unlike traditional models, GEE provides robust estimates of population‐averaged effects, even when the data exhibit intra‐subject correlation, making it ideal for analyzing the associations between physiological variables and outcomes measured at multiple time points. Data from each time point were combined into a single variable, resulting in a total of 115 values (from 23 individuals measured at 5 time points each). The significance level adopted was α ≤5%. Data were analyzed using IBM SPSS Statistics, Version 21.0 (IBM Corp., Armonk, NY, USA).

## 3 RESULTS

### 3.1 Physiological variables

The physiological variables SpO_2_ [*F*(4, 423.5) = 36.4, *p* < 0.001, *η* ^2^ₚ = 0.623] and heart rate (HR) [*F*(2, 353.8) = 5.235, *p* = 0.006, *η* ^2^ₚ = 0.192] showed significant changes over time, as determined by repeated‐measures GLM. Specifically, SpO_2_ decreased significantly during hypoxic exposure compared to baseline (*p* < 0.001), with a large effect size indicating that 62.3% of the variability in SpO_2_ was explained by the experimental condition. HR increased from baseline to the third (*p* = 0.02) and fourth hours (*p* < 0.001), with a moderate effect size explaining 19.2% of the variance. In total, 9% of participants met the diagnostic threshold for AMS (LLS ≥3 with headache present) at least once during the protocol. Descriptive statistics of all variables over time are presented in Table 1.

**TABLE 1:** Means and standard deviations of physiological, cognitive, and mood variables across five time points during hypoxic exposure.

	Before	1 h	2 h	3 h	4 h
Physiological variables					
HR (bpm)	73.2 (12.5)	78.3 (9.1)	77.0 (10.6)	79.2 (9.9)	81.0 (13.6)
Glucose (mg/dl)	82.0 (21.6)	91.4 (25.3)	84.5 (12.3)	78.5 (15.4)	76.3 (15.5)
Lactate (mmol/L)	1.8 (1.1)	2.6 (0.9)	2.2 (0.7)	2.4 (0.9)	2.1 (1.0)
SpO_2_ (%)	96.3 (1.8)	85.8 (4.1)	85.6 (4.0)	88.3 (4.0)	88.9 (3.7)
Lake Louise (score)	0.4 (0.6)	0.4 (0.7)	0.4 (0.9)	0.6 (1.2)	0.9 (1.5)
*Neurobehavioral variables*					
Behavioral attention					
Reaction time (ms)	255.4 (22.9)	259.3 (23.0)	258.7 (33.1)	261.0 (31.4)	253.2 (23.8)
Lapses (units)	0.4 (0.7)	0.4 (0.8)	0.2 (0.5)	0.3 (0.7)	0.1 (0.5)
Mood states					
Tension	2.0 (1.9)	0.6 (1.0)	0.4 (1.0)	0.6 (1.2)	0.5 (1.0)
Depression	0.3 (1.1)	0.1 (0.6)	0.1 (0.4)	0.2 (1.0)	0.2 (1.2)
Anger	1.1 (1.2)	0.8 (1.4)	0.6 (1.1)	0.8 (1.9)	0.8 (1.6)
Vigor	8.4 (2.2)	7.7 (3.4)	7.1 (3.4)	6.7 (3.6)	7.7 (3.5)
Fatigue	2.3 (2.2)	1.7 (2.1)	1.5 (1.5)	2.1 (2.8)	1.9 (2.7)
Confusion	0.7 (1.3)	0.4 (1.1)	0.4 (1.0)	0.3 (1.2)	0.4 (1.1)

*Note*: Values are expressed as mean (standard deviation) (*n* = 23).

### 3.2 Neurobehavioral measures

**FIGURE 1:**
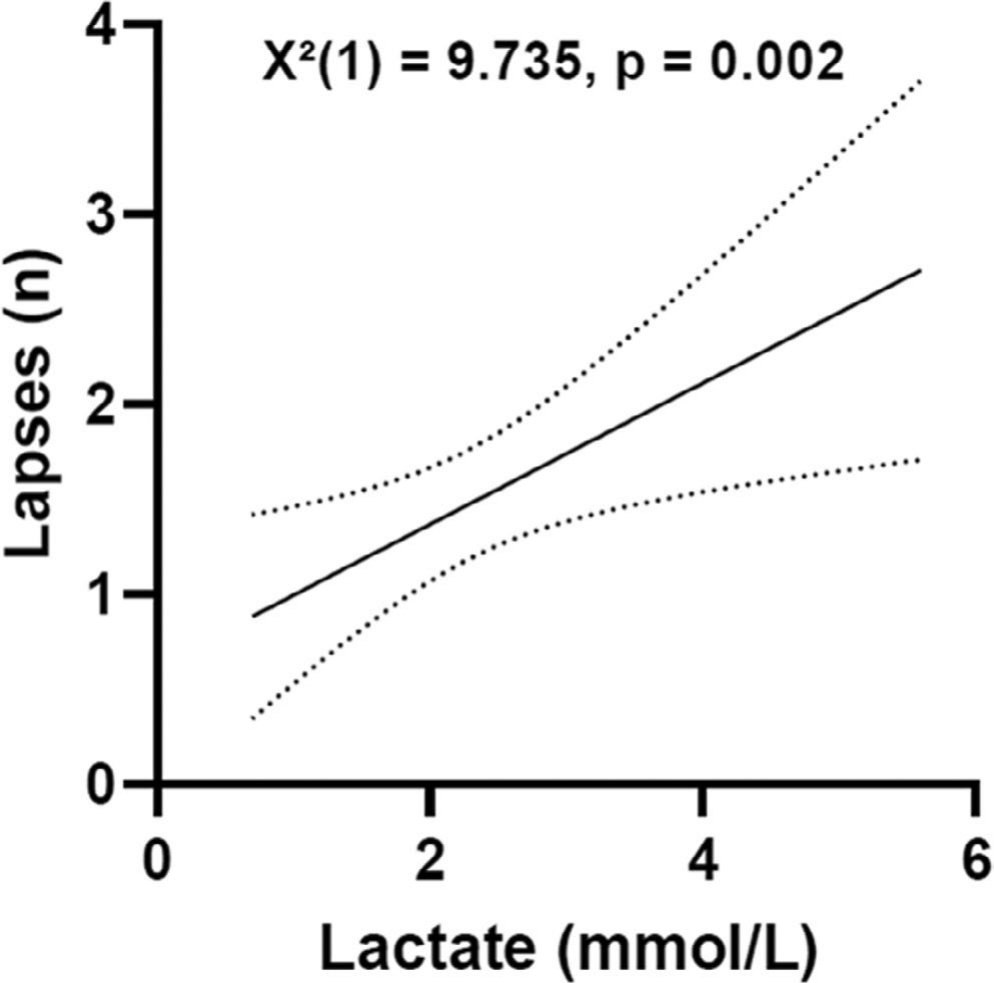
Relationship between blood lactate levels and lapses in reaction time. Scatter plot illustrating the number of reaction‐time lapses (*n*) as a function of blood lactate levels (mmol/L). The solid line represents the predicted trend from the Generalized Estimating Equations (GEE) model, with dotted lines indicating the 95% confidence interval. GEE analysis revealed a significant increase in lapses with higher lactate levels (Wald *χ* ^2^₁ = 9.735, *p* = 0.002, *N* = 111).

To assess possible cognitive effects of hypoxia, reaction time and the number of lapses (defined as responses >355 ms) were analyzed using repeated‐measures GLM. No significant changes over time were observed for either reaction time [*F*(2, 5469.8) = 2.494, *p* = 0.11] or lapses [*F*(2, 10.393) = 1.558, *p* = 0.23]. To further explore whether physiological responses could explain individual variability in performance, we performed a Generalized Estimating Equations (GEE) analysis. The results indicated that reaction time was not significantly associated with any physiological variable. However, the number of lapses was significantly associated with lactate levels [Wald *χ* ^2^₁ = 9.735; *p* = 0.002; *N* = 111], indicating that higher lactate concentrations predicted more lapses (see Table 2 and Figure 1).

**TABLE 2:** Results of the GEE analysis between Lapses and physiological variables.

Variable	*B* (95% CI)	*p*
HR	−0.001 (−0.009 to 0.007)	0.800
SpO_2_	−0.17 (−0.052 to 0.018)	0.346
Lactate	0.213 (0.079 to 0.348)	0.002*
Glucose	0.006 (0.000 to 0.012)	0.057

*Note*: The association between physiological variables and the number of lapses was examined through a GEE analysis including all values of each participant across five time points (baseline, 1, 2, 3, and 4 h), totaling 111 values for each variable.

Abbreviation: CI, confidence interval.

* Association between Lactate and Number of Lapses, *p* < 0.05.

### 3.3 Mood states

Mood states were assessed throughout the exposure period. Repeated‐measures GLM showed no significant time effects on mood dimensions. However, GEE analysis revealed significant associations between AMS symptoms and both Tension [Wald *χ* ^2^₁ = 21.106; *p* = 0.001; *N* = 115] and Fatigue [Wald *χ* ^2^₁ = 172.305; *p* < 0.001]. These results suggest that individuals experiencing more severe AMS symptoms also reported greater fatigue and tension levels (see Table 3).

**TABLE 3:** Results of the GEE analysis between BRUMS and Lake Louise Scores.

Variable	*B* (95% CI)	*p*
Tension	−0.118 (−0.169 to −0.068)	0.0001*
Depression	−0.216 (−0.433 to 0.002)	0.052
Anger	0.155 (−0.081 to 0.391)	0.197
Vigor	−0.038 (−0.144 to 0.067)	0.474
Fatigue	0.315 (0.268 to 0.362)	0.0001*
Confusion	0.074 (−0.109 to 0.256)	0.429

*Note*: The association between BRUMS dimensions and Lake Louise scores was examined through a GEE analysis including all values of each participant across five time points (baseline, 1, 2, 3, and 4 h), totaling 115 values for each variable. The statistical model included the variables Tension, Depression, Anger, Vigor, Fatigue, and Confusion.

* Association with increased Lake Louise symptoms.

## 4 DISCUSSION

The primary findings of this study indicate a significant association between elevated lactate levels and the number of lapses, as well as between mood states (particularly tension and fatigue) and Acute Mountain Sickness (AMS) symptoms. However, we observed no significant changes in reaction time, number of lapses, or mood dimensions over the four‐hour exposure to a simulated altitude of 4500 m in a normobaric chamber. These results partially align with those reported by Figueiredo et al. (2022), who found no effect of simulated altitude (3000–4050 m) on cognitive performance, but a marked impact on mood states after 20 h of exposure. This discrepancy suggests that cognition and mood may be affected at different stages during early acclimatization, despite concurrent physiological responses such as reduced SpO_2_ and elevated heart rate. Supporting this idea, a 31‐day hypobaric simulation of Everest ascent showed that increased anxiety was associated with faster reaction time, while mood dimensions such as tension, confusion, and hostility were negatively related to cognitive performance (Bolmont et al., 2000).

Previous research has shown that both altitude level and exposure duration critically influence cognitive function, and that sufficient acclimatization may even improve cognitive outcomes at high altitude (Bliemsrieder et al., 2022). Thus, the absence of significant changes in cognitive or mood performance in our study likely reflects the limited exposure time, which may not have been sufficient to elicit detectable impairments. Notably, early cognitive decline during acclimatization in other studies has been attributed, in part, to hypoxia‐induced sleep disruption (Figueiredo et al., 2022; Lemos et al., 2012), which was not assessed here.

The observed association between lactate and lapses may be explained by lactate's role in tissue acidosis, oxidative stress, and potential neurotoxicity under acute hypoxic conditions. Elevated lactate has been linked to impaired excitatory postsynaptic transmission and cognitive dysfunction (Wang et al., 2022). However, at high altitudes (2300–5700 m), increased lactate production may also serve adaptive purposes, acting as a neuronal energy substrate, promoting vasodilation, and triggering cellular signaling pathways involved in acclimatization (Proia et al., 2016).

Given the short exposure time (4 h), it is plausible that these neuroprotective adaptations had not yet occurred, contributing to fatigue and attentional lapses. Previous studies have directly linked fatigue symptoms to increased lapse frequency (Lee et al., 2010), and it is possible that longer exposure would allow lactate to assume its compensatory role in supporting brain function (Todd, 2014). Future studies should consider including a post‐exposure (“return‐to‐sea‐level”) assessment to evaluate recovery dynamics following acute hypoxic exposure.

The relationship between mood disturbances and AMS symptoms, particularly in the domains of tension and fatigue, reinforces the idea that psychological factors play a central role in AMS onset. This is consistent with a study involving 163 individuals aged 18–60 years exposed to 3700 m for 18–22 h, which identified depression, anger, fatigue, and confusion as key predictors of AMS (Bian et al., 2015).

In the present study, we employed the BRUMS scale, a short‐form mood assessment, and observed associations between AMS symptoms and the fatigue and depression subscales. In contrast, Niedermeier et al. (2017), using the State–Trait Anxiety Inventory (STAI), found no link between trait anxiety and AMS development after 12 h of normobaric hypoxia at 4500 m. This divergence may reflect methodological differences: BRUMS assesses multiple mood states, while the STAI focuses narrowly on anxiety. Moreover, the STAI was administered 37 months later, which may have introduced recall bias. Bolmont et al. (2000), who used both STAI and the Profile of Mood States (POMS) during a 31‐day hypobaric simulation, reported increases in anxiety and fatigue and reductions in vigor—but did not analyze AMS directly.

## 5 CONCLUSION

Our findings demonstrate that, although physiological parameters such as SpO_2_ and heart rate were significantly affected by acute exposure to simulated high altitude, reaction time and mood states remained stable over the 4‐h period. Importantly, higher lactate levels were associated with an increased number of lapses, indicating a potential relationship between metabolic stress and attentional performance. Additionally, mood dimensions, particularly tension and fatigue, were strongly associated with Acute Mountain Sickness (AMS) symptoms. These results highlight the combined influence of physiological and psychological factors in AMS development. The observed associations suggest that interventions targeting both metabolic and emotional responses to hypoxia, especially tension and fatigue, may be essential for improving adaptation and reducing AMS risk during early exposure to high altitude.

## AUTHOR CONTRIBUTIONS

**Ricardo Muller Bottura:** Conceptualization, data curation, formal analysis, investigation, writing–original draft. **Ambra Bisio:** Data analysis, writing–review and editing. **Debora Cristina Hipolide:** Supervision, project administration, writing–review and editing. All authors read and approved the final manuscript.

## FUNDING INFORMATION

This paper was funded by the Coordenação de Aperfeiçoamento de Pessoal de Nível Superior–Brasil (CAPES), Associação Fundo de Incentivo à Pesquisa (AFIP), and Fundação de Amparo à Pesquisa do Estado de São Paulo (FAPESP, 2014/27198‐8).

## CONFLICT OF INTEREST STATEMENT

The authors report no conflict of interest.
